# Structural analysis and evolution of specificity of the SUMO UFD E1-E2 interactions

**DOI:** 10.1038/srep41998

**Published:** 2017-02-06

**Authors:** Bing Liu, L. Maria Lois, David Reverter

**Affiliations:** 1 Institut de Biotecnologia i de Biomedicina, Departament de Bioquimica i Biologia Molecular, Serra Hunter Fellow, Universitat Autonoma de Barcelona, 08193 Barcelona, Bellaterra, Spain; 2 Center for Research in Agricultural Genomics-CRAG, Edifici CRAG-Campus UAB, Bellaterra 08193 Barcelona, Spain

## Abstract

SUMO belongs to the ubiquitin-like family (UbL) of protein modifiers. SUMO is conserved among eukaryotes and is essential for the regulation of processes such as DNA damage repair, transcription, DNA replication and mitosis. UbL modification of proteins occurs via a specific enzymatic cascade formed by the crosstalk between the E1-activating enzyme, the E2-conjugating enzyme and the E3-ligase. An essential discrimination step in all UbL modifiers corresponds to the interaction between E1 and E2 enzymes, which is mediated by the recruitment of the E2 to the UFD domain (Ubiquitin-Fold Domain) of the E1 enzyme. To gain insights in the properties of this interface, we have compared the structures of the complexes between E1 UFD domain and E2 in human and yeast, revealing two alternative UFD platforms that interact with a conserved E2. Comparative sequence analysis of the E1 UFD domain indicates that the E2 binding region has been conserved across phylogenetic closely related species, in which higher sequence conservation can be found in the E2 binding region than in the entire UFD domain. These distinctive strategies for E1-E2 interactions through the UFD domain might be the consequence of a high selective pressure to ensure specificity of each modifier conjugation system.

The post-translational modification pathway of proteins by UbLs (Ubiquitin-like modifiers) is characterized by the presence of specific enzymatic cascades (E1, E2 and E3 enzymes), which results in the formation of an isopeptidic bond between the UbL and the protein target^1^,^2^. A major characteristic of this process is the specificity provided between all components of the UbL pathway, resulting in the formation of protein-protein complementary interfaces^3^,^4^. SUMO (Small Ubiquitin-Like Modifier) is a UbL modifier that can alter the function of a myriad of target proteins inside the cell^5^, being involved in processes such as DNA damage repair, transcription, DNA replication and mitosis^1^,^6^,^7^.

The first specificity step in the pathway corresponds to the interaction of the UbL modifier with its particular E1-activating enzyme^8^,^9^. In the SUMO pathway, the E1-activating enzyme is a large multidomain heterodimer (Sae1-Uba2)^10^ that initiates the process by adenylation of the SUMO C-terminus and the subsequently formation of a thioester bond with the active-site cysteine residue of E1^11^. Next, the activated UbL-thioester is transferred to the active-site cysteine residue of the E2-conjugating enzyme (Ubc9 in SUMO), which represents a second specificity step in the pathway by the formation of the E1-E2 complex. Crystal structures of several E1-activating enzymes^12^,^13^,^14^,^15^ revealed the presence of a domain displaying an Ubiquitin-like Fold (UFD domain) in the E1-activating enzyme large subunit. The E1 UFD domain plays a major role in the binding of the E2 enzyme, providing specific contacts between E1 and E2 enzymes. This interaction was first observed in the crystal structure of the Nedd8 E1 in complex with E2^13^, showing the direct binding of E2 to the E1 UFD domain. Recently, the crystal structure of the thio-ester transfer intermediate of ubiquitin E1-E2 complex^15^ revealed a dual binding of E2 to the UFD domain and to the catalytic E1 Cys-domain, which occurs after an significant rotation of the UFD domain, providing the structural basis for the isoenergetic thio-ester transfer between the E1 and the E2 enzymes^15^. This interaction between E2 and the Cys domain of the E1 was proposed previously in the SUMO pathway by NMR analyses, although E1 UFD-E2 interactions display higher affinity (*K*_*d*_ = 1.2 μM)^16^ than E1 Cys-E2 interactions (*K*_*d*_ = 87 μM)^17^, supporting a major role of the E1 UFD domain in E2 recruitment.

Protein sequence variations in the E1 UFD domain of different UbL modifiers are quite significant, especially in the binding region to the E2 enzyme. All reported structures of UFD domains display an analogous β-grasp structure, and the interaction of the UFD domain with the E2-enzyme occurs through the same side of the β-sheet structure^13^,^14^,^15^,^18^,^19^,^20^. However, in all reported complex structures superposition of the E2 enzymes reveal distinct orientations of the UFD domain, which is a direct consequence of different contacts in each UbL system^15^,^18^. Conservation analysis according to sequence alignments showed that yeast and human SUMO UFD domains display little sequence homology (17% sequence identity), and it is even lower considering only the binding region to Ubc9. Notwithstanding this low conservation, both proteins can efficiently interact with a highly conserved surface in their cognate Ubc9. In this scenario, the identification of the molecular determinants that mediate E1 UFD and E2 interactions in evolutionary distant organisms cannot rely on sequence homology analysis. Instead, the elucidation of these molecular determinants requires specific structural studies of the interaction.

Here we present a detailed structural comparison analysis of the two complexes between SUMO E1 UFD domain and Ubc9 from yeast^21^ and human. We also present a novel structure of the human complex solved in a different space group than the recently deposited^22^. Our results indicate that human and yeast UFD domains interact with a conserved surface of Ubc9, in each case by maintaining the same chemical character of the interface contacts despite the lack of sequence homology. Sequence alignment of these two E2 binding region discloses unique consensus motifs that have been maintained across species from the same kingdom (in *Metazoa*) or in the same order (in *Saccharomycetales*). Phylogenetic and homology analysis revealed that the region involved in Ubc9 binding displays a slightly higher conservation degree than the UFD domain between phylogenetically closely related organisms, although it also displays higher variability, highlighting the relevance of this interface in the protein-protein specificity for each type of UbL modification.

## Results

### Complex between human SUMO E1 UFD domain and SUMO E2

The interaction between the ubiquitin-fold domain (UFD) of the E1-activating enzyme and the E2-conjugating enzyme has been revealed as a crucial discrimination step in the conjugation pathway of UbL modifiers^14^,^18^,^23^,^24^. The interface between E1 and E2 enzymes is unique and is required to confer specificity between cognate enzymes of each UbL conjugation pathway. Notably, differences in the interface are more significant in the UFD domain than in the E2-conjugating enzyme, which is a highly conserved enzyme. Within the same UbL family, evolutionary distant species display a low degree of sequence conservation between E1 UFD domains. In SUMO pathway, yeast and human UFD domains were shown to display only a 17% sequence identity^25^. To get structural insights for the different interaction between yeast and human, we have determined the crystal structure of the complex between human E1 Uba2 UFD domain and human E2 Ubc9 at 2.2 Å resolution and compared this structure to the analogous complex in yeast (PDB code 3ONG)^25^. During the preparation of the manuscript, another structure of the human E1 UFD-Ubc9 was also published in a different crystallographic space group, supporting our results^22^ (PDB ID 4W5V).

Human E1 ubiquitin-fold domain (UFD) was designed based on the structure of the full-length human SAE1-SAE2 E1-activating enzyme (PDB code 1Y8Q)^24^. We alternatively prepared a UFD construct including the C-terminal flexible extension, however this longer UFD was unstable and displayed proteolysis after complex formation with Ubc9. Both native gel electrophoresis and gel filtration chromatography indicated the formation of the complex between human E1 UFD domain and Ubc9 (see Supplementary Fig. S1). After initial unfruitful crystallization trials with the purified complex, a lysine methylation protocol was conducted to induce crystallization. Suitable crystals diffracted beyond 2.2 Å resolution, contained one complex of UFD/Ubc9 per asymmetric unit and belonged to the tetragonal space group system (Table 1), which is different to the monoclinic crystals recently deposited^22^ (PDB code 4W5V).

An overall structural comparison of free and bound Ubc9 structures displayed little variation (0.71 Å rmsd Cα deviation), although differences were observed in the binding region to UFD, displaying rmsd Cα deviations between 1.50 Å and 3.66 Å in the residues forming the β1-β2 loop (Lys30 to Met36). Similarly, free and bound E1 UFD structures are almost identical (overall 0.72 Å rmsd deviation). According to the PISA server^26^ the human complex buries a surface of 1493 A^2^ and involves 26 and 19 residues of UFD and Ubc9, respectively, which is comparable to the analogous complex in yeast (1557 A^2^ interface)^25^.

### Yeast and human interface comparison

**Figure 1:**
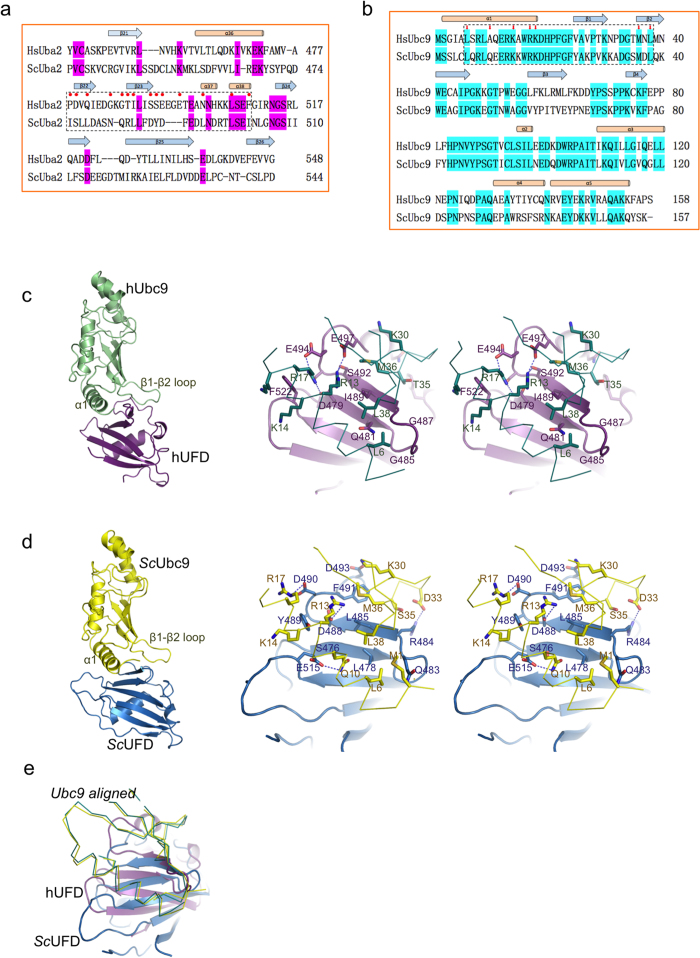
Structural alignment and comparison of the interfaces between UFD and Ubc9 from human and yeast complexes. (**a**) Structural alignment of the UFD domains from yeast (*ScUba2*) and human (*HsUba2*). Red circles indicate contact residues to Ubc9. Dotted rectangle represents the binding region to Ubc9. (**b**) Structural alignment of yeast and human Ubc9. Small red arrows indicate contact residues to UFD. Dotted rectangle represents the binding region to UFD. Secondary structure is depicted above sequence. (**c**) *Left*, ribbon representation of the complex of human Ubc9 and the UFD domain. *Right*, stereo representation of the interface residues between Ubc9 (green line) and UFD (purple ribbon). Major contacts are labeled and represented in stick configuration. (**d**) *Left*, ribbon representation of the complex of yeast Ubc9 and the UFD domain. *Right*, stereo representation of the interface residues between Ubc9 (yellow line) and UFD (blue ribbon). Major contacts are labeled and represented in stick configuration. (**e**) Structural superposition of Ubc9 in the human and yeast complex with UFD.

In contrast to the conservation proposed according to sequence homology between human and yeast UFD, 17% of identity, this homology is even lower, 11%, when a structural alignment is performed (Fig. 1a). Interestingly, differences between yeast and human are increased, 7% of sequence identity, when comparing only the residues forming the interface of the E1 UFD domain with Ubc9, which are basically formed by a different set of residues capable to interact with a conserved Ubc9 surface (Fig. 1b). However, despite this low sequence homology between yeast and human UFD, the interaction occurs through the same surface^22^,^25^, forming an interdigitated complex between the α1 helix and the β1-β2 loop of Ubc9 that sits on the β-sheet surface of UFD (Fig. 1c). As a consequence of this low conservation, structural superposition of Ubc9 reveals a rotation of the E1 UFD domain between both complexes (Fig. 1e). Different orientations of UFD domains in complex with E2 can also be observed in other UbL systems, as revealed by the structures of ubiquitin and Nedd8 UFD in complex with E2 (Uba1-Ubc4 complex PDB code 4II2; and Uba3-Ubc12, pdb code 2NVU refs 13 and 15). All these structures suggest plasticity in the UFD interface that has evolved to specifically interact with its cognate E2-conjugating enzyme in each UbL pathway.

**Figure 2:**
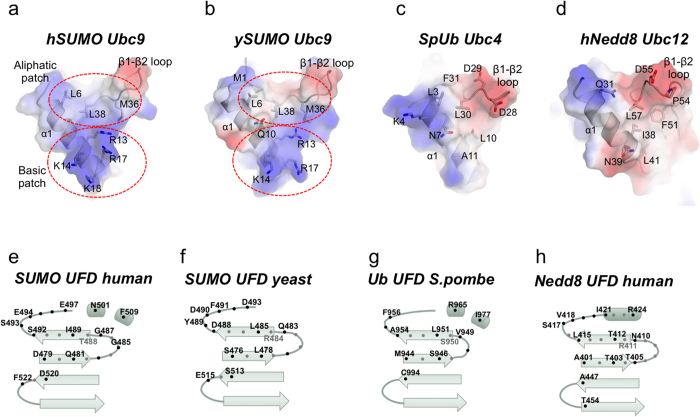
Comparison of the UFD-E2 interface from different UbL systems. (**a**) Transparent electrostatic representation of the interface of human SUMO E2 (*hSUMO Ubc9*) with the UFD domain. Major contacts are labeled and represented in stick configuration. Basic and aliphatic surface patches are indicated by dotted circles. (**b**) Transparent electrostatic representation of the interface of yeast SUMO E2 (*ySUMO Ubc9*) with the UFD domain. Major contacts are labeled and represented in stick configuration. Basic and aliphatic surface patches are indicated by dotted circles. (**c**) Transparent electrostatic representation of the interface of *S.pombe* ubiquitin E2 (*SpUb Ubc4*) with the UFD domain. Major contacts are labeled and represented in stick configuration. (**d**) Transparent electrostatic representation of the interface of human Nedd8 E2 (*hNedd8 Ubc12*) with the UFD domain. Major contacts are labeled and represented in stick configuration. (**e**) Schematic representation of the human SUMO E1 UFD domain contacts with the E2 enzyme. (**f**) Schematic representation of the yeast SUMO E1 UFD domain contacts with the E2 enzyme. (**g**) Schematic representation of the *S.pombe* ubiquitin E1 UFD domain contacts with the E2 enzyme. (**h**) Schematic representation of the human Nedd8 E1 UFD domain contacts with the E2 enzyme. Black and grey spots indicate the orientation of the side chain in the structure regarding the β-sheet plane.

The binding surface of Ubc9 in yeast and human is highly conserved, and is basically formed by similar backbone and side-chain interactions in the α1 helix and the β1-β2 loop of Ubc9 (yeast and human Ubc9 share 56% sequence identity). Specific side-chain interactions in Ubc9 include a hydrophobic patch formed by Leu6, Met36, and Leu38, and a basic patch formed by Arg13, Lys14, Arg17 and Lys18 (Fig. 2a,b). Interestingly, all these conserved residues in Ubc9 engage specific contacts with the non-conserved UFD surfaces of yeast and human. A major contact difference in Ubc9 corresponds to Ala10, which is substituted by Gln10 in yeast, forming a polar interaction with Glu515 of the yeast UFD domain (Fig. 1d). Previous point mutational analysis on these two patches of Ubc9, namely the basic α1 helix and the hydrophobic β1-β2 loop, showed impairment in the binding to the E1 activating enzyme, indicating a major role of these two regions in the transfer of SUMO between E1 and E2 proteins^25^,^27^.

**Figure 3:**
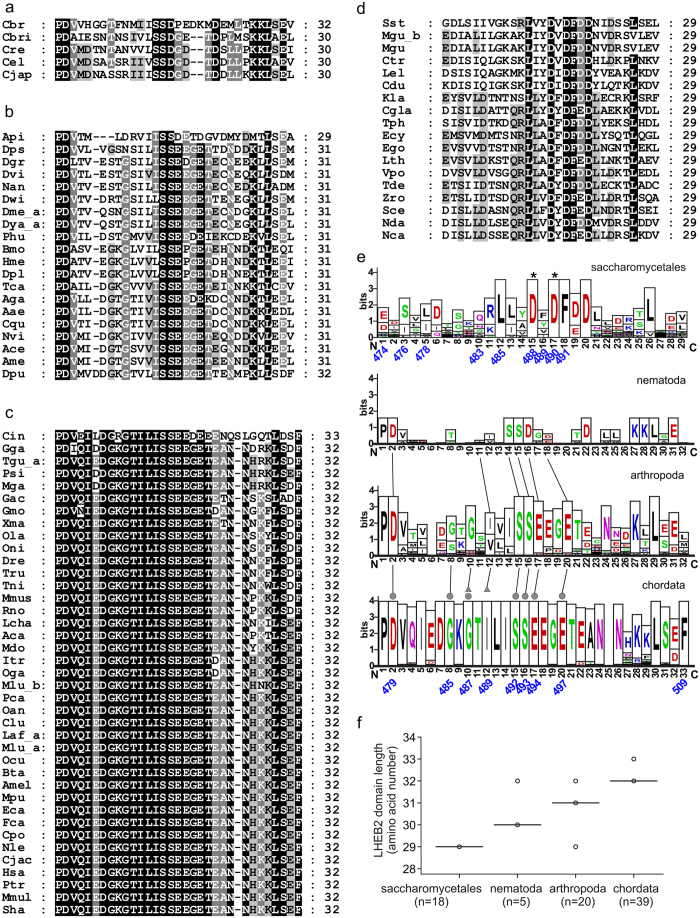
Conservation analysis of Sae2 LHEB2 domain in metazoan and Saccharomycetales. Amino acid sequence alignment of Sae2 LHEB2 domain orthologs from nematoda (**a**), arthopoda (**b**), chordate (**c**) and saccharomycetales (**d**). Residue shading correspond to 95% (white letter and dark background), 75% (white letter and gray background), and 55% (black letter and light gray background) of sequence identity in (**a**,**b** and **c**). In the case of saccharomycetales, the shading types correspond to 90%, 70% and 50% of sequence identity, respectively. Metazoan multiple sequence alignments of LHEB2 sequences were performed using Clustal Omega software and including yeast Uba2 (NP_010678) as outlier. Saccharomycetales multiple sequence alignments of LHEB2 sequences were performed using Muscle software and including human Sae2 (Q9UBT2) as outlier. (**e**) Graphical representation of LHEB2 domain consensus sequences determined from amino acid sequence alignments shown in (**a**,**b**,**c** and **d**). The overall height of the stack indicates the sequence conservation at that position, while the symbol height within the stack indicates the relative frequency of each amino acid within that position. The positions of yeast Sae2 residues involved in Ubc9 interaction according to the previously resolved structure (3ONG) are indicated in blue below the sequences graph. Asterisks indicate residues shown to have a major contribution to E1-E2 interactions in mutagenesis analysis^25^. The positions of human Sae2 residues involved in Ubc9 interactions according to the resolved structure are indicated in blue below the chordata consensus sequence graph. Grey circles indicate residues establishing contacts with Ubc9 α1-helix, while grey triangles indicate residues interacting with Ubc9 residues located at the Ubc9 β1β2-loop. Conserved residues across phyla are indicated by lines. (**f**) Distribution of LHEB2 sequence length displayed by orthologs within each phylogenetic group analyzed was plotted on a box plot graph. Data points are represented by circles. Outliers are represented by dots. The number of data points analyzed in each phylogenetic group is indicated below the x-axis.

The UFD interacting surface is extended and mostly formed by residues emanating from β22 and β23 strands and connecting loops (Fig. 1c,d). A general feature of this interaction, widespread so far in all characterized UbL pathways, is the interaction through the same β-sheet surface (Fig. 2). But in contrast to the Ubc9, the region involved in UFD is poorly conserved between yeast and human. For simplicity, we have designated this region as *Low Homology region involved in E2 Binding 2* (LHEB2). The LHEB2 can be divided in two regions, each establishing interactions with the basic and hydrophobic patches of Ubc9 (Fig. 2a,b). The first contact region in human UFD is composed by Asp479, Ser492, Ser493, Glu494 and Glu497, which engage polar and charged contacts with the basic patch of Ubc9, composed by α1 helix residues Arg13 and Arg17. The LHEB2 in human is highly conserved in all *metazoan* species analyzed (see later in Fig. 3). In contrast, in yeast the basic patch of Ubc9 interacts with Asp488, Tyr489 and Asp490, which is also a highly conserved sequence in all *Saccharomycetales* species analyzed (see later in Fig. 3). Interestingly, in the human structure Phe522 is buried in an aliphatic pocket formed by Arg17, Lys14 and Lys18 (see Fig. 1c), whereas in yeast a similar Ubc9 pocket buries Tyr489 (see Fig. 1d), a residue located at the center of the LHEB2 sequence instead of the LHEB2 C-terminal position that occupies its human counterpart.

The second contact region of UFD interacts with the hydrophobic patch of Ubc9. This interface is composed by backbone and side chain interactions emanating from the β22-β23 connecting loop and β23 strand of UFD. In human Ubc9 Leu6 interacts with the β22-β23 loop formed by Gly485 and Gly487 (Fig. 1c). In contrast, in yeast, the composition and length of this loop is different and Ubc9 Leu6 interacts with Leu478 (Fig. 1d). In this region we can observe the highest structural homology between human and yeast. In human three backbone hydrogen bonds are formed between Gly487 and Ile489 with Ubc9 Met36 and Asn37, but only two in yeast, between Leu485 and Ubc9 Met36. Additionally, the side chain of Ile489 in human (or Leu485 in yeast) is buried in both cases in the Ubc9 hydrophobic pocket formed by Met36 and Leu38 (Fig. 1c,d). Finally, it is worth mentioning that the specific contacts established by yeast Arg484 and Phe491 are absent in metazoan sequences but highly conserved in *Saccharomycetales*.

### Interface comparison with other UbL E1-E2 complexes

Comparison of the UFD-E2 interfaces in ubiquitin and Nedd8 (Uba1-Ubc4 complex, PDB code 4II2; and Uba3-Ubc12, pdb code 1Y8X)^13^,^15^ indicate that only the hydrophobic patch in Ubc9 is partially conserved in the ubiquitin E2 (Ubc4), formed in this instance by Leu3 and Leu30, which can be aligned with Ubc9 Leu6 and Leu38 (Fig. 2). However, the basic patch in the α1 helix of Ubc9, formed by Arg13, Lys14, Arg17 and Lys18, represents a specific feature of the SUMO pathway and are not present neither in Nedd8 (Ubc12) nor in ubiquitin (Ubc4), which are replaced by acidic and aliphatic residues. Ubc4 and Ubc12 also display a shorter β1-β2 loop compared to Ubc9 (Fig. 2). These differences in the E2-conjugating enzyme between UbLs modifiers result in the presence of non-complementary surfaces with E1 UFD domains, which are indeed the basis for the enzyme specificity among each UbL pathway. For instance, SUMO E1 UFD domain contains specific polar contacts (Ser492, Ser493, Glu494 and Glu497 in human or Asp488, Asp490 and Asp493 in yeast) to interact with the basic patch of Ubc9, however, in ubiquitin and Nedd8 these positions are substituted by aliphatic residues (Ala954 and Phe956 in ubiquitin or Leu415, Val418 and Ile421 in Nedd8) (Fig. 2).

As mentioned before, human Ile489 and yeast Leu485 adopt a similar conformation and engage identical backbone hydrogen bonds with the β1-β2 loop of Ubc9. Interestingly, despite the lack of sequence conservation, the equivalent residues in the ubiquitin UFD domain, namely Ser950-Leu951, also engage analogous backbone hydrogen bonds contacts with the β1-β2 loop of the E2 enzyme (Ubc4)^15^. Thus this backbone interaction represents a unique conserved structural element maintained in distant UbL systems such as SUMO and ubiquitin. This interaction does not occur in Nedd8 (Ubc12-Uba3, pdb code 1Y8X ref. 13), in which the E2 enzyme sits across a similar region of the UFD domain but with a different angle compared to ubiquitin, and the contacts between both UbL systems are poorly conserved and thus not complementary^18^,^15^.

### Evolutionary conservation of the SUMO E1 UFD interfaces

**Figure 4:**
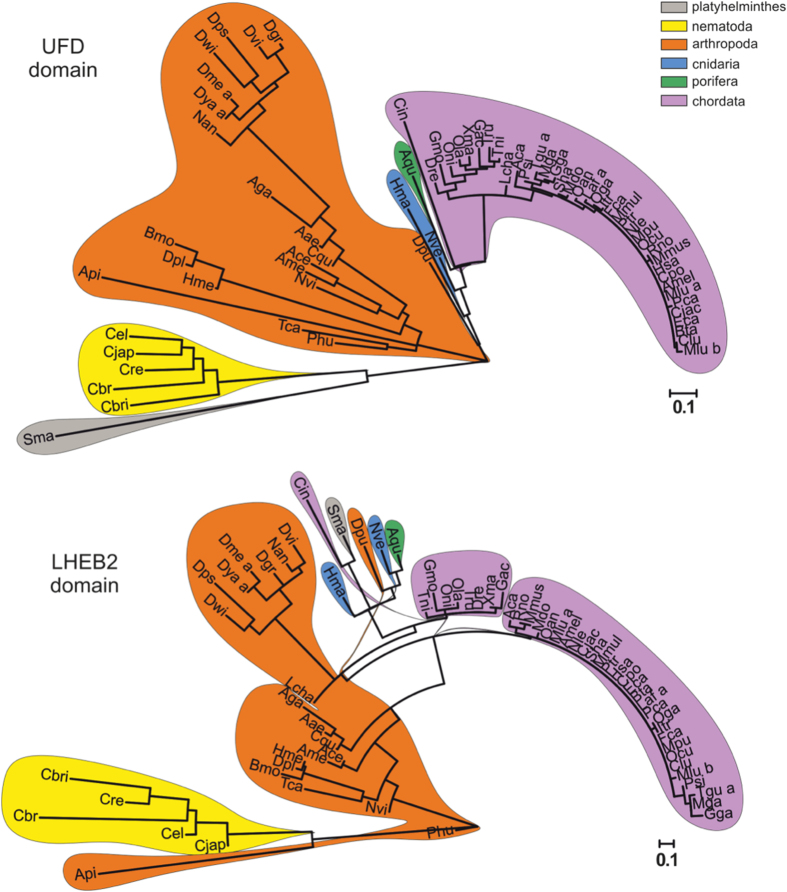
Phylogenetic analysis of UFD and LHEB2 domains from Metazoa and Saccharomycetales. Maximum likelihood phylogenetic trees depicting the evolutionary relationships among 86 Sae2 UFD (**a**) or Sae2 LHEB2 (**b**) domain sequences from 68 metazoa species species using sequence alignments shown in Fig. S3 (UFD) and Fig. 3 (LHEB2). Sequences belonging to the same phylum are enclosed in colored areas. Tree scales are shown below each tree.

Structural comparison between human and yeast indicate that SUMO UFD domains are composed by a different set of residues that can engage productive interactions with a conserved E2 enzyme. In order to evaluate the biological relevance of these two alternative structural interfaces involved in E2 interactions, we have analyzed the conservation of the LHEB2 sequence of the E1 UFD domains across species. In human, the LHEB2 region is comprised by residues between Pro478 and Phe509 (Figs 1a and 3e), whilst in yeast is composed by residues between Pro472 and Ile502 (Figs 1b and 3e). We searched for human E1 UFD domain orthologs at the EggNOG database and focused on metazoan and fungal species, for which structural information of the E1 UFD-E2 interactions is available. Since fungal E1 displayed a high level of sequence divergence, we focused on species belonging to the *Saccharomycetales* order, which include *Saccharomyces cerevisae*. The phylogenetic analyses of both domains, E1 UFD and LHEB2, show that clustering of the E1-UFD sequences in the phylogenetic tree reflects the taxonomical relationships of the species represented (Fig. 4a). On the contrary, when the same analysis was performed with the LHEB2 domain, tree distribution is not consistent with taxonomic lineages (Fig. 4b), suggesting that this region presents higher variability than the UFD domain where is contained.

In addition, we analyzed the distribution of homology between pairs. *Saccharomycetales* UFD or LHEB2 sequences were compared with the corresponding human (conservation to outlier) or yeast sequences (conservation within the group). Similarly, metazoan UFD or LHEB2 sequences were compared with the corresponding yeast (conservation to outlier) or human sequences (conservation within the group). The homology pair distributions were plotted onto box plots (Supplemental Fig. S2). In general, when sequences were compared with an outlier, in all groups except in *Saccharomycetales*, the median of the obtained distribution was higher in the LHEB2 sequence analysis than in the UFD. At the same time, the box length, whose limits indicate the 25th and 75th percentiles, is higher for the LHEB2 than for the UFD homology pair distributions, suggesting that this region also presents higher variability. These results support the phylogenetic analyses indicating that, in general, the LHEB2 sequence is more conserved than the UFD domain within each evolutionary group, although the individual sequences contained in each group display higher variability. Similar results were obtained when sequences were compared with a reference sequence within each group, *Saccharomycetales* or metazoan.

The homology analysis of the residues involved in Ubc9 contacts are even higher conserved and minor differences were identified among phyla. In chordates, the highest conserved group, all the contacts described in the human structure are present in all species analyzed within this phylum (Fig. 3c). In arthropoda, sequence comparison also display little divergence, but in this case variations can be found in the composition of the β22-β23 connecting loop of UFD, presenting different loop lengths but still conserving Gly485 and Gly487 in many species of the phylum (Fig. 3b). In nematode, the five species analyzed also display conservation in the major E2 contact residues, but in contrast to chordate and arthropoda, the β12-β13 connecting loop displays little homology and Gly485 and Gly487, present in chordata and arthropoda, have been substituted (Fig. 3a).

Therefore, all major specific UFD contacts with Ubc9 are basically conserved in *metazoa*, including contacts with the hydrophobic and the basic patches of the Ubc9 surface. For instance, human Asp479, Ser492, Ser493, Glu494 and Glu497 (occasionally replaced by aspartic), which interact with Ubc9 Arg13 and Arg17, are highly conserved in all species analyzed. Similarly, the hydrophobic interaction of the human UFD Ile489, which interacts with Ubc9 Met36 and Leu38, is also conserved but can be occasionally substituted by valine in some species. The major differences in the UFD interface in *metazoa* are located in the β22-β23 connecting loop, formed in human by Gly485 and Gly487, which interact with Ubc9 Leu6. Whereas in chordate this connecting loop is highly conserved, in arthropod and particularly in nematode, this loop displays different lengths and amino acid composition. It is worth mentioning here that the extension and composition of this particular loop in UFD domains is highly divergent between E1-activating enzymes specific to different UbL modifiers, suggesting that this domain has evolved to interact with its cognate E2-enzyme.

In contrast to *metazoa*, sequence comparison of the E2 binding region of the yeast UFD domain with members of the *Fungi* kingdom is highly diverse, and further structural analyses will be required for establishing the molecular basis of E1-E2 interactions in those divergent groups. In the order of *Saccharomycetales*, the major specific contacts described in the structure of *S. cerevisiae* UFD-Ubc9 complex^25^ are conserved. In this instance, as shown before in the structural comparison, the consensus binding sequence in *Saccharomycetales* is completely different to *metazoan*. Yeast UFD residues Asp488, Asp490 and Asp493, which interact with the basic patch of Ubc9, are highly conserved in *Saccharomycetales* (Fig. 3d,e), as well as Leu478, Leu485 and Phe491, which interact with the hydrophobic patch of Ubc9. Other specific contacts in the structure, such as Arg484 and Tyr489, are replaced by residues with similar chemical properties, such as lysine for Arg484 and, phenylalanine, isoleucine or valine for Tyr489 (Fig. 3d,e).

In the essential SUMO conjugation pathway, protein-protein interactions have evolved to maintain specificity of the modifier and the targets. The SUMO conjugating enzyme constitutes the link between modifier specificity, which is selected by the E1 activating enzyme, and the protein substrate specificity, mediated by the cooperation between the E2 and the E3 ligase enzymes. In the E1-E2 interactions, a region in the E1 UFD domain, the LHEB2 sequence, plays a major role in E2 recruitment to the E1. In evolutionary distant groups, the LHEB2 sequences are poorly conserved according to sequence homology and length. On the contrary, in closely related phylogenetic groups, the conservation of the LHEB2 sequence is higher, highlighting the relevance of this interaction between E1 and E2 structures. We speculate that these distinctive strategies for E1-E2 interactions through the E1 UFD domain are the consequence of a high selective pressure to ensure modifier specificity. Future structural analyses of other evolutionary distant groups, such as plants or protozoa, will most probably uncover novel molecular determinants mediating E1-E2 interactions.

## Conclusions

In summary, structural comparison of yeast and human SUMO UFD-E2 complexes and sequence alignment of SUMO UFD domains, reveal the presence of at least two complementary types of interfaces, which are conserved across species from the same kingdom (*metazoan*) or in the case of the *Fungi* kingdom, in the same order (*Saccharomycetales*). Despite the low level of sequence homology in the UFD domains among these distant species of different kingdoms, these two types of interfaces maintain the structural and chemical properties necessary to interact with a conserved E2 binding surface. Structural and sequential comparisons have also revealed at least two different types of consensus sequences in the E1 UFD domain, which we named LHEB2 sequences, that can complement the conserved surface in the E2 enzyme. Interestingly, sequence conservation in the E2 binding region is higher than in the overall UFD domain, suggesting the presence of an evolutionary pressure to maintain the contacts with the E2-conjugating enzyme, which are essential for the correct function of each UbL pathway.

## Materials and Methods

### Protein expression and purification

Expression constructs were generated by a standard PCR-based cloning method. The full length human Ubc9 and E1 UFD domain (residues 447–547) were cloned to pET28a tagged with 6x His at the N-terminal. *Escherichia coli* BL21(DE3) plysS containing the expression vector were grown in Luria Bertani medium with chloramphenicol (17 μg/mL) and kanamycine (50 μg/mL) at 37 °C until the OD600 reached to 0.8. Expression was induced by 0.1 mM IPTG, followed by overnight culturing at 28 °C. Recombinant proteins were purified by nickel-nitrilotriacetic acid agarose resin (Qiagen) and dialyzed against 250 mM NaCl, 20 mM Tris-HCl (pH 8.0), 1 mM β-mercaptoethanol in the presence of thrombin protease overnight at 4 °C to remove the 6x His tag. Proteins were further purified by gel filtration chromatography on a Superdex75 column (GE Healthcare), which was pre-equilibrated in 250 mM NaCl, 20 mM Tris-HCl pH 7.5, 1 mM β-mercaptoethanol.

### Protein complex preparation and methylation

Ubc9 and UFD complex was made by mixing equimolar amounts of proteins and purified by gel filtration chromatography using a Superdex75 column. Ubc9 and UFD were co-eluted in a single peak and confirmed by SDS-PAGE. After gel filtration, the complex was dialyzed against 250 mM NaCl, 50 mM HEPES pH 7.5, 1 mM β-mercaptoethanol for lysine methylation based on a published strategy^28^. In brief, borane-dimethylamine complex (Sigma-Aldrich) and formaldehyde (Sigma-Aldrich) were sequentially added into protein solution and incubated overnight at 4 °C. The methylation reaction was stopped by a final gel filtration chromatography on a Superdex75 column pre-equilibrated in 200 mM NaCl, 20 mM Tris-HCl (pH 7.5), 1 mM β-mercaptoethanol. Purified protein complex was concentrated to 30 g/L using an Amicon Ultra-10 K ultrafiltration device (Millipore) prior to crystallization.

### Crystallization and data collection

Crystals were grown by the sitting-drop vapor diffusion method by mixing the protein complex (30 g/L) with an equal volume of reservoir solution containing 16% PEG6000 (w/v), 100 mM MES pH 6.5, and 5% MPD (v/v), at 18 °C. Crystals appeared after 24 hours and continued to grow to full size in one week. Big crystals were soaked in mother liquor supplemented with gradually increasing concentration of 5%, 10%, 20% (v/v) MPD for 60 seconds each time and flash frozen in liquid nitrogen. Diffraction data were collected to 2.20 Å resolution at ALBA synchrotron in Barcelona (BL13-XALOC beamline). The crystals belong to the space group P4_3_212 and the unit cell has a dimension of a = 129.61 Å, b = 129.61 Å, and c = 66.60 Å. Data were processed with XDS^29^ and scaled, reduced, and further analyzed using CCP4^30^. More details are shown in Table 1.

### Structure determination and refinement

The structure was determined by molecular replacement method using the full length human Ubc9 (protein data bank code 1U9B) as a search model for one molecule in the asymmetric unit in PHASER^31^. Initial electron density was manually improved to build up the final model using Coot^32^, and the refinement was performed using Phenix^33^. Refinement statistics are shown in Table1. The structure has been deposited in the PDB data bank with the code 5FQ2.

### Phylogenetic sequence comparison

Human Sae2 orthologs were search in EggNOG database (http://eggnogdb.embl.de/#/app/home) and sequences from *Metazoa* and *Saccharomycetales* were selected for homology analysis. Detailed information about sequence homology analysis methods is indicated in figure legends. Briefly, when sequences were highly divergent, multiple sequence alignments were performed using Muscle tool (http://www.ebi.ac.uk/Tools/msa/muscle/). When sequence displayed higher conservation level, homology analyses were performed using Clustal Omega program (http://www.clustal.org/omega/). Phylogenetic distances were calculated by the maximum likelihood method and the JTT model included in the Seaview v4 software package^34^, and unrooted trees exported. Phylogenetic trees were drawn using the online iTOL software (http://itol.embl.de/). Consensus sequences were calculated using WebLogo software (http://weblogo.berkeley.edu/)^35^. Multiple sequence alignments were edited, analyzed and shaded using GeneDoc software^36^ (http://iubio.bio.indiana.edu/soft/molbio/ibmpc/genedoc-readme.html). Data distribution was plotted on box plots using “BoxPlotR: a web-tool for generation of box plots”.

## Additional Information

**Accession codes:** The crystal structure of the complex was deposited in the PDB data bank with the code 5FQ2.

**How to cite this article:** Liu, B. *et al*. Structural analysis and evolution of specificity of the SUMO UFD E1-E2 interactions. *Sci. Rep.* **7**, 41998; doi: 10.1038/srep41998 (2017).

**Publisher's note:** Springer Nature remains neutral with regard to jurisdictional claims in published maps and institutional affiliations.

## Supplementary Material

Supplementary Information

## Figures and Tables

**Table 1:** Summary of crystallographic analysis.

Data collection	
Beamline	ALBA-XALOC
Space group	P4_3_2_1_2
Wavelength (Å)	0.97946
Resolution (Å)	46.44–2.20 (2.32–2.20)*
a, b, c (Å)	129.61, 129.61, 66.60
α, β, γ (°)	α = β = γ = 90
Unique reflections	29309
Data redundancy	10.9 (11.0)
R_merge_	0.043 (0.602)
I/σ	26.4 (4.3)
Completeness (%)	99.8 (98.4)
**Refinement**	
Resolution (Å)	46.44–2.20
Unique reflections	29254
R_work_/R_free_	0.22/0.24
Number of all atoms	2100
Number of waters	38
RMSD bond (Å)/Angle (°)	0.008/1.11
Average B factor (protein/water)	60.69/56.26
**Ramachandran plot**	
Favored (%)	97.64
Allowed (%)	2.36
Disallowed (%)	0.00

^*^Highest resolution shell is shown in parenthesis.
