# Supplementary material for: Structural analysis and evolution of specificity of the SUMO UFD E1-E2 interactions
Source: Sci Rep. 2017 Feb 6;7:41998. doi: 10.1038/srep41998 (PMC5292753; doi:10.1038/srep41998)
Supplement: Supplementary Information [file srep41998-s1.pdf]

Structural analysis and evolution of specificity of the SUMO UFD E1-E2 interactions

Bing Liu[1], L. Maria Lois[2*] and David Reverter[1*]

[1]Institut de Biotecnologia i de Biomedicina and Departamento de Bioquimica i Biologia Molecular, Universitat Autonoma de Barcelona, 08193 Barcelona, Bellaterra, Spain.

[2]Center for Research in Agricultural Genomics-CRAG. Edifici CRAG-Campus UAB, Bellaterra 08193 Barcelona, Spain

*To whom the correspondence should be addressed: David Reverter, Universitat Autonòma de Barcelona, 08193 Bellaterra, Barcelona, Spain, Ph: +34 93 5868955, FAX: (93)5812011, E-mail: david.reverter@uab.cat and L. Maria Lois, Center for Research in Agricultural Genomics-CRAG, 08193 Bellaterra, Barcelona, Spain, Ph: +34 93 5636600 ext.3215, Fax. +34 93 5636601, email: maria.lois@cragenomica.es.

Supplementary Information

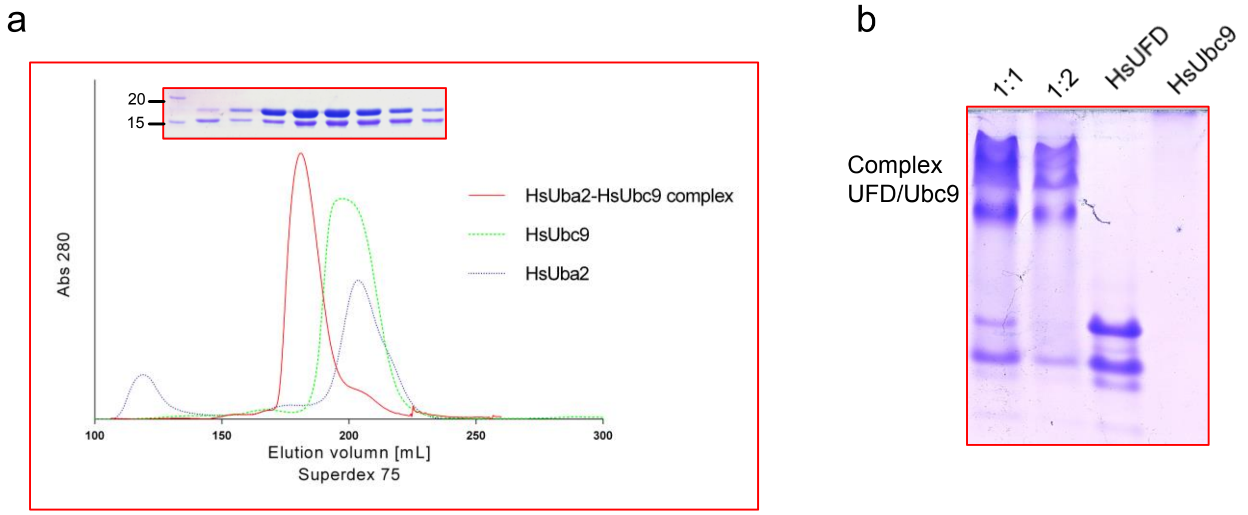

Supplementary Figure S1. Purification of the complex between human E1 UFD domain and Ubc9. (a) Size exclusion chromatography profiles of human Ubc9, human E1 UFD domain and the complex with a buffer containing 100mM NaCl, 20mM Tris 8, 1mM BME. Inset, SDS-PAGE of the peak fractions. (b) Native gel electrophoresis of the complex formation between human UFD domain and human Ubc.

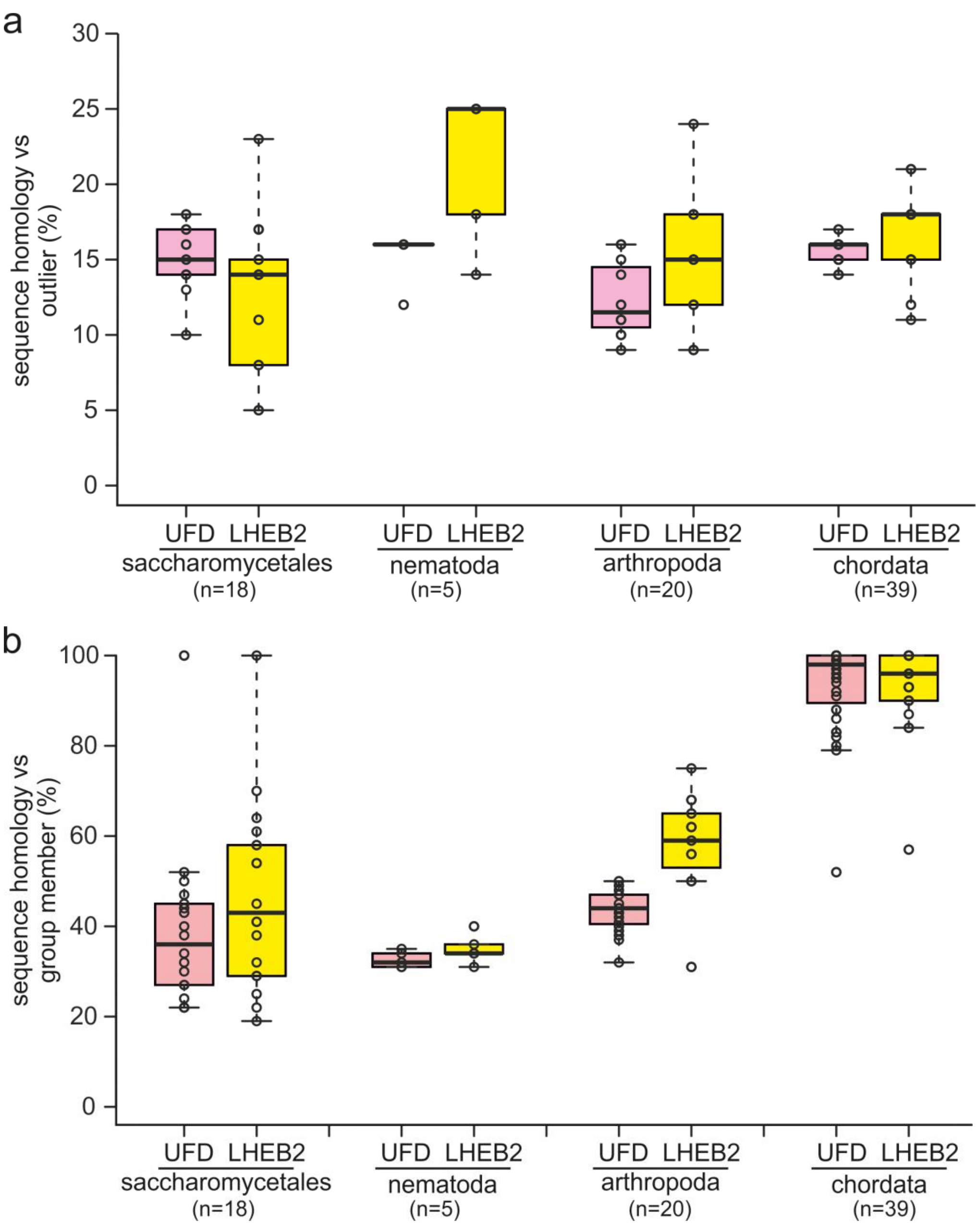

Supplementary Figure S2. Analysis of pair homology distribution between UFD or LHEB2 sequences within each phylogenetic group analyzed. (a) Distribution of identity percentage of UFD (pink boxes) or LHEB2 (yellow boxes) domains between each sequence in the analyzed group and an outlier sequence (human, in the case of saccharomycetales, and yeast, in the case of nematoda, arthropoda and chordata). (b) Distribution identity percentage of UFD (pink boxes) or LHEB2 (yellow boxes) domains between each sequence in the analyzed group and a sequence from the same group (yeast, in the case of saccharomycetales, and human, in the case of nematoda, arthropoda and chordata). Center lines show the medians; box limits indicate the 25th and 75th percentiles as determined by R software; whiskers extend 1.5 times the interquartile range from the 25th and 75th percentiles, outliers are represented by dots; data points are plotted as open circles.

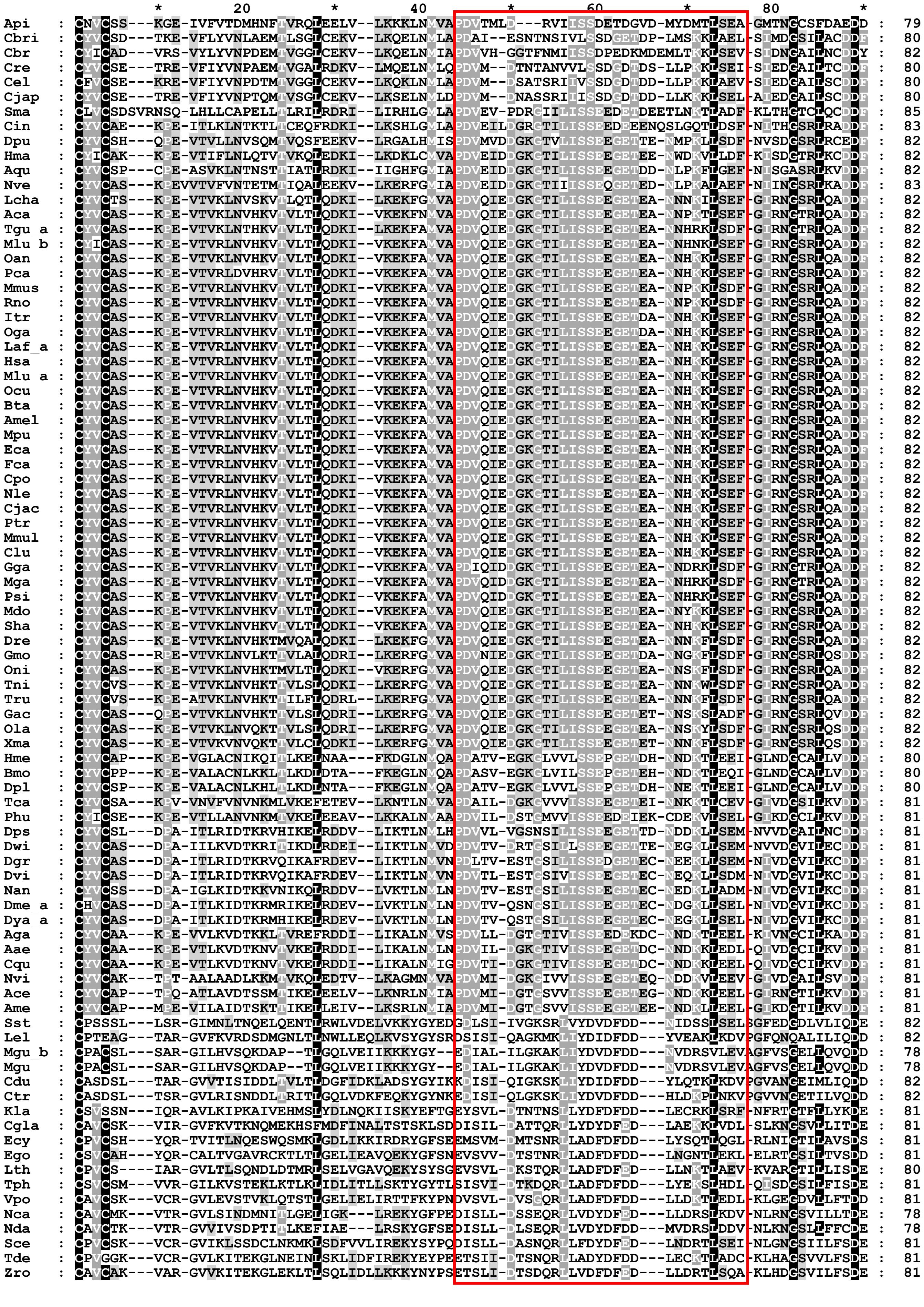

* 20 * 40 * 60 * 80 *
Api : CNVCSS---KGE-IVFVTDMHNFTVRQLEELV--LKKKLNMVAPDVTMLD---RVIISSDETDGVD-MYDMTLSEA-GMTNGCSFDAEDD : 79
Cbri : CYVCSD---TKE-VFLYVNLAEMTLSGLCEKV--LKQELNMLAPDAI--ESNTNSIVLSSDGETDP-LMSKKLAEL-SIMDGSILACDDF : 80
Cbr : CYICAD---VRS-VYLYVNPDEMTVGGLCEKV--LKQELNMIAPDVVH-GGTFNMIISSDPEDKMDEMLTKKLSEV-SIDNGAILNCDDY : 82
Cre : CYVCSE---TRE-VFIYVNPAEMTVGALRDKV--LMQELNMLQPDVM--DTNTANVVLSDGDTDS-LLPKKLSEI-SIEDGAILTCDDF : 80
Cel : CFVCSE---KRE-VFIYVNPDTMTVGGLCEKV--LKQKLNMLAPDVM--DSATSRIIVSSDGDTDD-LLPKKLAEV-SIEDGAILSCDDF : 80
Cjap : CYVCSE---TRE-VFIYVNPTQMTVSGLCEKV--LKSELNMLDPDVM--DNASSRIIISSDGDTDD-LLKKKLSEL-AIEDGAILSCDDF : 80
Sma : CLVCSDSVRNSQ-LHLLCAPELLTLRILRDRI--LIRHLGMLAPDVEV-PDRGIILISSEDETDEETLNKTLADF-KLTHGTCLQCDDF : 85
Cin : CYVCAE---KPE-ITLKLNTKTLTCEQFRDKI--LKSHLGMLAPDVEILDGRGTILISSEDEEENQSLGQTLDSF-NITHGSRLRADDF : 83
Dpu : CYVCSH---QPE-VTVLLNVSQMTVQSFEEKV--LRGALHMISPDVMVDDGKGTVLISSEGETTE-NMPKLLSDF-NVSDGSRLRCEDF : 82
Hma : CYICAK---KPE-VTIFLNLQTVTVKQLEDKI--LKDKLCMVAPDVEIDDGKGTILISSEGETEE-NWDKVLLDF-KISDGTRLKCDDF : 82
Aqu : CYVCSP---CPE-ASVKLNTNSTTIATLQDKI--IIGHFGMIAPDVEIDDGKGTILISSEGETDD-NLPKFLGEF-NISGASRLKVDDF : 82
Nve : CYVCAS---KPEVVTVFVNTETMTIQALEEKV--LKERFGMIAPDVEIDDGKGTIIISSEQETED-NLPKALAEF-NIINGSRLKADDF : 83
Lcha : CYVCTS---KPE-VTVKLNVSKVTLQTLQDKI--LKEKFGMVAPDVQIEDGKGTILISSEGETEA-NNNKILSEF-GIRNGSRLQADDF : 82
Aca : CYVCAS---KPE-VTVKLNVHKVTVLTLQDKI--VKEKFNMVAPDVQIEDGKGTILISSEGETEA-NNPKTLSEF-GIRNGTRLQADDF : 82
Tgu_a : CYVCAS---KPE-VTVKLNTHKVTVLTLQDKI--LKEKFAMVAPDVQIEDGKGTILISSEGETEA-NNHRKLSDF-GIRNGTRLQADDF : 82
Mlu_b : CYICAS---KPE-VTVRLNVHKVTVLTLQDKI--VKEKFAMVAPDVQIEDGKGTILISSEGETEA-NNHNKLSEF-GIRNGSRLQADDF : 82
Oan : CYVCAS---KPE-VTVKLNVHKVTVLTLQDKI--VKEKFAMVAPDVQIEDGKGTILISSEGETEA-NNHKKLSEF-GIRNGSRLQADDF : 82
Pca : CYVCAS---KPE-VTVRLDVHRVTVLTLQDKI--VKEKFAMVAPDVQIEDGKGTILISSEGETEA-NNHKKLSEF-GIRNGSRLQADDF : 82
Mmus : CYVCAS---KPE-VTVRLNVHKVTVLTLQDKI--VKEKFAMVAPDVQIEDGKGTILISSEGETEA-NNPKKLSDF-GIRNGSRLQADDF : 82
Rno : CYVCAS---KPE-VTVRLNVHKVTVLTLQDKI--VKEKFAMVAPDVQIEDGKGTILISSEGETEA-NNPKKLSDF-GIRNGSRLQADDF : 82
Itr : CYVCAS---KPE-VTVRLNVHKVTVLTLQDKI--VKEKFAMVAPDVQIEDGKGTILISSEGETDA-NNHKKLSEF-GIRNGSRLQADDF : 82
Oga : CYVCAS---KPE-VTVRLNVHKVTVLTLQDKI--VKEKFAMVAPDVQIEDGKGTILISSEGETDA-NNHKKLSEF-GIRNGSRLQADDF : 82
Laf_a : CYVCAS---KPE-VTVRLNVHKVTVLTLQDKI--VKEKFAMVAPDVQIEDGKGTILISSEGETEA-NNHKKLSEF-GIRNGSRLQADDF : 82
Hsa : CYVCAS---KPE-VTVRLNVHKVTVLTLQDKI--VKEKFAMVAPDVQIEDGKGTILISSEGETEA-NNHKKLSEF-GIRNGSRLQADDF : 82
Mlu_a : CYVCAS---KPE-VTVRLNVHKVTVLTLQDKI--VKEKFAMVAPDVQIEDGKGTILISSEGETEA-NNHKKLSEF-GIRNGSRLQADDF : 82
Ocu : CYVCAS---KPE-VTVRLNVHKVTVLTLQDKI--VKEKFAMVAPDVQIEDGKGTILISSEGETEA-NNHKKLSEF-GIRNGSRLQADDF : 82
Bta : CYVCAS---KPE-VTVRLNVHKVTVLTLQDKI--VKEKFAMVAPDVQIEDGKGTILISSEGETEA-NNHKKLSEF-GIRNGSRLQADDF : 82
Amel : CYVCAS---KPE-VTVRLNVHKVTVLTLQDKI--VKEKFAMVAPDVQIEDGKGTILISSEGETEA-NNHKKLSEF-GIRNGSRLQADDF : 82
Mpu : CYVCAS---KPE-VTVRLNVHKVTVLTLQDKI--VKEKFAMVAPDVQIEDGKGTILISSEGETEA-NNHKKLSEF-GIRNGSRLQADDF : 82
Eca : CYVCAS---KPE-VTVRLNVHKVTVLTLQDKI--VKEKFAMVAPDVQIEDGKGTILISSEGETEA-NNHKKLSEF-GIRNGSRLQADDF : 82
Fca : CYVCAS---KPE-VTVRLNVHKVTVLTLQDKI--VKEKFAMVAPDVQIEDGKGTILISSEGETEA-NNHKKLSEF-GIRNGSRLQADDF : 82
Cpo : CYVCAS---KPE-VTVRLNVHKVTVLTLQDKI--VKEKFAMVAPDVQIEDGKGTILISSEGETEA-NNHKKLSEF-GIRNGSRLQADDF : 82
Nle : CYVCAS---KPE-VTVRLNVHKVTVLTLQDKI--VKEKFAMVAPDVQIEDGKGTILISSEGETEA-NNHKKLSEF-GIRNGSRLQADDF : 82
Cjac : CYVCAS---KPE-VTVRLNVHKVTVLTLQDKI--VKEKFAMVAPDVQIEDGKGTILISSEGETEA-NNHKKLSEF-GIRNGSRLQADDF : 82
Ptr : CYVCAS---KPE-VTVRLNVHKVTVLTLQDKI--VKEKFAMVAPDVQIEDGKGTILISSEGETEA-NNHKKLSEF-GIRNGSRLQADDF : 82
Mmul : CYVCAS---KPE-VTVRLNVHKVTVLTLQDKI--VKEKFAMVAPDVQIEDGKGTILISSEGETEA-NNHKKLSEF-GIRNGSRLQADDF : 82
Clu : CYVCAS---KPE-VTVRLNVHKVTVLTLQDKI--VKEKFAMVAPDVQIEDGKGTILISSEGETEA-NNHKKLSEF-GIRNGSRLQADDF : 82
Gga : CYVCAS---KPE-VTVRLNVHKVTVLTLQDKI--VKEKFAMVAPDIQIDDGKGTILISSEGETEA-NNDRKLSDF-GIRNGTRLQADDF : 82
Mga : CYVCAS---KPE-VTVRLNVHKVTVLTLQDKI--VKEKFAMVAPDVQIDDGKGTILISSEGETEA-NNHRKLSDF-GIRNGTRLQADDF : 82
Psi : CYVCAS---KPE-VTVKLNVHKVTVLTLQDKI--VKEKFAMVAPDVQIDDGKGTILISSEGETEA-NNHRKLSEF-GIRNGSRLQADDF : 82
Mdo : CYVCAS---KPE-VTVKLNVHKVTVLTLQDKI--VKEKFAMVAPDVQIEDGKGTILISSEGETEA-NNYKKLSEF-GIRNGSRLQADDF : 82
Sha : CYVCAS---KPE-VTVKLNVHKVTVLTLQDKI--VKEKFAMVAPDVQIEDGKGTILISSEGETEA-NNHKKLSEF-GIRNGSRLQADDF : 82
Dre : CYVCAS---KPE-VTVKLNVHKTMVQALQDKI--LKEKFGMVAPDVQIEDGKGTILISSEGETEA-NNNKFLSDF-GIRNGSRLQADDF : 82
Gmo : CYVCAS---RPE-VTVKLNVLKTTVLALQDRI--LKERFGMVAPDVNIEDGKGTILISSEGETDA-NNGKFLSDF-GIRNGSRLQSDDF : 82
Oni : CYVCAS---KPE-VTVKLNVHKTMVLTLQDKI--VKERFGMVAPDVQIEDGKGTILISSEGETEA-NNSKFLSDF-GIRNGSRLQADDF : 82
Tni : CYVCVS---KPE-VTVKLNVHKTTVLSLQDKI--LKERFGMVAPDVQIEDGKGTILISSEGETEA-NNNKWLSDF-GIRNGSRLQADDF : 82
Tru : CYVCVS---KPE-ATVKLNVHKTTILFLQDRL--LKERFGMVAPDVQIEDGKGTILISSEGETEA-NNNKFLSDF-GIRNGSRLQADDF : 82
Gac : CYVCAS---QPE-VTVKLNVHKTTVLSLQDKI--LKERFGMVAPDVQIEDGKGTILISSEGETET-NNSKSLADF-GIRNGSRLQVDDF : 82
Ola : CYVCAS---KPE-VTVKLNVQKTTVLSLQDRI--LKERFGMVAPDVQIEDGKGTILISSEGETEA-NNSKYLSDF-GIRNGSRLQSDDF : 82
Xma : CYVCAS---KPE-VTVKVNVQKTTVLCLQDKI--LKERFGMVAPDVQIEDGKGTILISSEGETET-NNNKFLSDF-GIRNGSRLQSDDF : 82
Hme : CYVCAP---KPE-VGLACNIKQITLKELNAA---FKDGLNMQAPDATV-EGKGLVVLSSEPGETDH-NNDKTLEEI-GLNDGCALLVDDF : 80
Bmo : CYVCPP---KPE-VALACNLKKLTLKDLDTA---FKEGLNMQAPDASV-EGKGLVILSSEPGETEH-NNDKTLEQI-GLNDGCALLVDDF : 80
Dpl : CYVCSP---KPE-VALACNLKHLTLKDLNTA---FKEGLNMQAPDATV-EGKGLVVLSSEPGETDH-NNEKTLEEI-GLNDGCALLVDDF : 80
Tca : CYVCSA---KPV-VNVFVNVNKMLVKEFETEV--LKNTLNMVAPDAIL-DGKGVVVISSEGETEI-NNKKTLCEV-GIVDGSILKVDDF : 81
Phu : CYICSE---KPE-VTLLANVNKMTVKELEEAV--LKKALNMAAPDVIL-DSTGMVVISSEDEIEK-CDEKVLSEL-GIKDGCLLKVDDF : 81
Dps : CYVCSL---DPA-ITLRIDTKRVHIKELRDDV--LIKTLNMLHPDVVL-VGSNSILISSEGETTD-NDDKLLSEM-NVVDGAILNCDDF : 81
Dwi : CYVCAS---DPA-IILKVDTKRITIKDLRDEI--LIKTLNMVDPDVTV-DRTGSILLSSEGETTE-NEGKLLSEM-NVVDGVILECDDF : 81
Dgr : CYVCAS---DPA-ITLRIDTKRVQIKAFRDEV--LIKTLNMVNPDLTV-ESTGSILISSDGETEC-NEEKLLSEM-NIVDGVILKCDDF : 81
Dvi : CYVCAS---DPA-ITLRIDTKRVQIKAFRDEV--LIKTLNMVNPDVTL-ESTGSIVISSEGETEC-NEQKLLSDM-NIVDGVILKCDDF : 81
Nan : CYVCSS---DPA-IGLKIDTKKVNIKQLRDDV--LVKTLNMLNPDVTV-ESTGSILISSEGETEC-NEDKLLADM-NIVDGVILKCDDF : 81
Dme_a : CHVCAS---DPA-ITLKIDTKRMRIKELRDEV--LVKTLNMLNPDVTV-QSNGSILISSEGETEC-NDGKLLSEL-NIVDGVILKCDDF : 81
Dya_a : CYVCAS---DPA-ITLKIDTKRMHIKELRDEV--LVKTLNMLNPDVTV-QSTGSILISSEGETEC-NEGKLLSEL-NIVDGVILKCDDF : 81
Aga : CYVCAA---KPE-VVLKVDTKKLTVREFRDDI--LIKALNMVSPDVLL-DGTGTIVISSEDEKDC-NDKTLEEL-KIVNGCILKADDF : 81
Aae : CYVCAA---KPE-VTLKVDTKNVTVKELRDDI--LIKALNMLNPDVIL-DGKGTIVISSEGETDC-NDKKLEDL-QIVDGCILKVDDF : 81
Cqu : CYVCAA---KPE-VTLKVDTKNVTVKELRDDI--LIKALNMIGPDVTI-DGKGTIVISSEGETDC-NDKKLEEL-QIVDGCILKVDDF : 81
Nvi : CYVCAK---TPT-AALAADLKKMTVKQLEDTV--LKAGMNMVAPDVMI-DGKGIVVISSEGETEQ-NDDKVLEEV-GIVDGAILSVDDF : 81
Ace : CYVCAP---TPQ-ATLAVDTSSMTIKELEELV--LKNRLNMIAPDVMI-DGTGSVVISSEGETEG-NDKKLEEL-GIRNGTILKVDDF : 81
Ame : CYVCAP---MPE-VILAIDTSKTTIKELLEIV--LKSRLNMIAPDVMI-DGTGSVVISSEGETEE-NDKKLEEL-GIKDGTILKVDDF : 81
Sst : CPSSSL---LSR-GIMNLTNQELQENTLRWLVDELVKKYGYEDGDLSI-IVGKSRLVYDVDFDD---NIDSSLSELSGFEDGDLVLIQDE : 82
Lel : CPTEAG---TAR-GVFKVRDSDMGNLTLNWLLEQLKVSYGYSRDSISI-QAGKMKLIYDIDFDD---YVEAKLKDVPGFQNQALILIQDD : 82
Mgu_b : CPACSL---SAR-GILHVSQKDAP--TLGQLVEIIKKKYGY--EDIAL-ILGKAKLIYDVDFDD---NVDRSVLEVAGFVSGELLQVQDD : 78
Mgu : CPACSL---SAR-GILHVSQKDAP--TLGQLVEIIKKKYGY--EDIAL-ILGKAKLIYDVDFDD---NVDRSVLEVAGFVSGELLQVQDD : 78
Cdu : CASDSL---TAR-GVVTISIDDLTVLTLDGFIDKLADSYGYIKKDISI-QIGKSKLIYDIDFDD---YLQTKLKDVPGVANGEIMLIQDD : 82
Ctr : CASDSL---TSR-GVLRISNDDLTRITLGQLVDKFEQKYGYNKEDISI-QLGKSKLIYDVDFDD---HLDKPLNKVPGVVNGETILVQDD : 82
Kla : CSVSSN---IQR-AVLKIPKAIVEHMSLYDLNQKIISKYEFTGEYSVL-DTNTNSLLYDFDFDD---LECRKLSRF-NFRTGTFLLYKDE : 81
Cgla : CAVCSK---VIR-GVFKVTKNQMEKHSFMDFINALTSTSKLSDDISIL-DATTQRLLYDYDFED---LAEKKLVDL-SLKNGSVLLITDE : 81
Ecy : CPVCSH---YQR-TVITLNQESWQSMKLGDLIKKIRDRYGFSEEMSVM-DMTSNRLLADFDFDD---LYSQTLQGL-RLNIGTILAVSDS : 81
Ego : CSVCAH---YQR-CALTVGAVRCKTLTLGELIEAVQEKYGFSNEVSVV-DTSTNRLLADFDFDD---LNGNTLEKL-ELRTGSILTVSDD : 81
Lth : CPVCS----IAR-GVLTLSQNDLDTMRLSELVGAVQEKYSYSGEVSVL-DKSTQRLLADFDFED---LLNKTLAEV-KVARGTILLISDE : 80
Tph : CSVCSM---VVR-GILKVSTEKLKTLKLIDLITLLSKTYGYTLSISVI-DTKDQRLLADFDFDD---LYEKSLHDL-QISDGSILFISDE : 81
Vpo : CAVCSK---VCR-GVLEVSTVKLQTSTLGELIELIRTTFKYPNDVSVL-DVSGQRLLADFDFDD---LLDKTLEDL-KLGEGDVLLFTDD : 81
Nca : CAVCMK---VTR-GVLSINDMNITLGELIGK---LREKYGFPEDISLL-DSSEQRLLVDYDFED---LLDRSLKDV-NLRNGSVILLTDE : 78
Nda : CAVCTK---VTR-GVVIVSDPTITLKEFIAE---LRSKYGFSEDISLL-DLSEQRLLVDYDFDD---MVDRSLDDV-NLKNGSLLFFCDE : 78
Sce : CPVCSK---VCR-GVIKLSSDCLNKMKLSDFVVLIREKYSYPQDISLL-DASNQRLLFDYDFED---LNDRTLSEI-NLGNGSIILFSDE : 81
Tde : CPVGGK---VCR-GVLKITEKGLNEINLSKLIDFIREKYEYPEETSII-DTSNQRLLADYDFDD---LECKTLADC-KLHAGSVVLFSDE : 81
Zro : CAVCAK---VAR-GVVKITEKGLEKLTLSQLIDLLKKKYNYPSETSLI-DTSDQRLLVDFDFED---LLDRTLSQA-KLHDGSVILFSDE : 81

```
                      100         *       120         *       140         *
Api    : HQNFR-------VKIIA-------------------------YDKE----KSGEEDLDFEILNN- : 107
Cbri   : MQDFG-------VKVFV-------------------------CKNS------NFKGDQFEIVRE- : 106
Cbr    : MQELE-------LKLFI-------------------------RSAD------HLKGDQFEVARE- : 108
Cre    : MQDMV-------LKLFI-------------------------RRAN------HLRGDNFEIARS- : 106
Cel    : QQEME-------IKLFI-------------------------KKGD------RLAGDDFEVARS- : 106
Cjap   : QQDMV-------LKLFI-------------------------RRQN------DLKGDEFKILRE- : 106
Sma    : RQDFT-------IRLILSCISVELANKLPTTSDLSTLNLNLSSRLEEQLTSEELQSETWRIVGD- : 142
Cin    : LQNYD-------IVVNI-------------------------LHEE----N-LPDDMIFQVSET- : 110
Dpu    : HQEYD-------LNITI-------------------------AHRD----Q-LEDNREFSIIAD- : 109
Hma    : LQNFE-------IAITL-------------------------RDKK----D-INVDQLFFLEGE- : 109
Aqu    : LQNYQ-------LILNL-------------------------FHSD----EALSDEKEFEVISD- : 110
Nve    : LQNYE-------LVINI-------------------------KHRT----D-LETDQEFEVEGD- : 110
Lcha   : LQDYT-------LLINV-------------------------LNSE----D-LEKDVEFEVVGD- : 109
Aca    : LQDYT-------LLINV-------------------------LHCE----E-LAKDVEFEVVGD- : 109
Tgu_a  : LQDYT-------LLINV-------------------------LHSE----D-LEKDVEFEVVGD- : 109
Mlu_b  : LQDYT-------LLINI-------------------------LHSE----D-LGKDIEFEVVGD- : 109
Oan    : LQDYT-------LLINL-------------------------LHSE----D-LGKDVEFEVVGD- : 109
Pca    : LQDYT-------LLINI-------------------------LHSE----D-LGKDVEFEVVGD- : 109
Mmus   : LQDYT-------LLINI-------------------------LHSE----D-LGKDVEFEVVGD- : 109
Rno    : LQDYT-------LLINI-------------------------LHSE----D-LGKDVEFEVVGD- : 109
Itr    : LQDYT-------LLINI-------------------------LHSE----D-LEKDVEFEVVGD- : 109
Oga    : LQDYT-------LLINI-------------------------LHSE----D-LGKDVEFEVVGD- : 109
Laf_a  : LQDYT-------LLINI-------------------------LHSE----D-LAKDVEFEVVGD- : 109
Hsa    : LQDYT-------LLINI-------------------------LHSE----D-LGKDVEFEVVGD- : 109
Mlu_a  : LQDYT-------LLINI-------------------------LHSE----D-LGKDVEFEVVGD- : 109
Ocu    : LQDYT-------LLINI-------------------------LHSE----D-LGKDVEFEVVGD- : 109
Bta    : LQDYT-------LLINI-------------------------LHSE----D-LGKDVEFEVVGD- : 109
Amel   : LQDYT-------LLINI-------------------------LHSE----D-LGKDVEFEVVGD- : 109
Mpu    : LQDYT-------LLINI-------------------------LHSE----D-LGKDVEFEVVGD- : 109
Eca    : LQDYT-------LLINI-------------------------LHSE----D-LGKDVEFEVVGD- : 109
Fca    : LQDYT-------LLINI-------------------------LHSE----D-LGKDVEFEVVGD- : 109
Cpo    : LQDYT-------LLINI-------------------------LHSE----D-LGKDVEFEVVGD- : 109
Nle    : LQDYT-------LLINI-------------------------LHSE----D-LGKDVEFEVVGD- : 109
Cjac   : LQDYT-------LLINI-------------------------LHSE----D-LGKDVEFEVVGD- : 109
Ptr    : LQDYT-------LLINI-------------------------LHSE----D-LGKDVEFEVVGD- : 109
Mmul   : LQDYT-------LLINI-------------------------LHSE----D-LGKDVEFEVVGD- : 109
Clu    : LQDYT-------LLINI-------------------------LHSE----D-LGKDIEFEVVGD- : 109
Gga    : LQDYT-------LLINV-------------------------LHSE----D-LEKDVEFEVVGD- : 109
Mga    : LQDYT-------LLINV-------------------------LHSE----D-LEKDVEFEVVGD- : 109
Psi    : LQDYT-------LLINV-------------------------LHSE----D-LEKDVEFEVVGD- : 109
Mdo    : LQDYT-------LLINV-------------------------LHSD----D-LEKDVEFEVVGD- : 109
Sha    : LQDYT-------LLINV-------------------------LHSD----D-LEKDVEFEVVGD- : 109
Dre    : LQDYT-------LLVNV-------------------------IHSE----E-LEKDVEFEVVGD- : 109
Gmo    : LQDYT-------LLINV-------------------------LHAE----D-LEKDVEFEVVGD- : 109
Oni    : LQDYT-------LLINV-------------------------LHVE----D-LERDVEFEVVGE- : 109
Tni    : LQDYT-------LLVNV-------------------------LHTE----E-LERDVEFEVIGE- : 109
Tru    : LQDYT-------LLVNV-------------------------LHTE----E-LERDVEFEVIGD- : 109
Gac    : LQDYA-------LLVNV-------------------------LHTE----E-LERDVEFEVVGE- : 109
Ola    : LQDYT-------LLINV-------------------------LHTE----E-LERDVEFEVVGE- : 109
Xma    : LQDYT-------LLINV-------------------------LHTE----E-LERDVEFEVVGE- : 109
Hme    : LQNYE-------VRVRL-------------------------QQDD--------DEKSWKLITD- : 104
Bmo    : LQNYE-------VRVRL-------------------------QQED--------EENSWRLVTD- : 104
Dpl    : LQNYE-------VRVRL-------------------------QQED--------EEKTWRLVTD- : 104
Tca    : LQNYE-------LIINV-------------------------NQYE----AKEKDDPPFKFIAN- : 109
Phu    : LQNYE-------LTVYV-------------------------NHYE-----APKDEPDFKIIAD- : 108
Dps    : HQNYS-------LSVII-------------------------SHFD----A-ERDDSLFEVSAD- : 108
Dwi    : HQNYK-------LRIII-------------------------NHFD----A-DREDNLFEVVAD- : 108
Dgr    : FQNYE-------LSIII-------------------------AHFD----A-EREDVLFEVIAD- : 108
Dvi    : FQSYE-------LSIII-------------------------AHFD----A-DREDVLFEVIAD- : 108
Nan    : FQNYE-------LSIII-------------------------SHFD----S-ERDEAVFEVVAD- : 108
Dme_a  : FQNYE-------LSIII-------------------------SHFD----A-ERDENLFEVVAD- : 108
Dya_a  : FQNYE-------LSIII-------------------------SHFD----A-ERDENLFEVVAD- : 108
Aga    : LQNYE-------LSITV-------------------------LHKE----A-GREEAPFEIVAD- : 108
Aae    : VQNYE-------LTVTV-------------------------IHKD----P-GRDESSFDIVAD- : 108
Cqu    : VQNYE-------LTVTI-------------------------VHKD----P-ARDEPSFDIVAD- : 108
Nvi    : LQNYS-------LKVTV-------------------------VHRE--KPLPNSDEPEFVITAD- : 111
Ace    : QQNYS-------LTVYI-------------------------VYREKPDPK-DQDSPQFLILAD- : 112
Ame    : QQNYS-------LTITI-------------------------IYRE--RPSLKGDSPDFLILAD- : 111
Sst    : NDELE------NLELYI-------------------------TVVN-------------EPTTEK : 103
Lel    : DDLLE------KLELLI-------------------------NVVDPGPEGDYNGIVLTKLPSV- : 115
Mgu_b  : NDELE------NLEFYI-------------------------SLGD---------------KNE- :  96
Mgu    : NDELE------NLEFYI-------------------------SLGD---------------KNE- :  96
Cdu    : ADELE------NLELYL-------------------------NIKD--------DTVEFR----- : 103
Ctr    : DDQLE------NLQLYL-------------------------SIVD-----EQCDLELPDLKLR- : 110
Kla    : EMEDG-AQLRQSIEFYL-------------------------EFVP---ENSGEDLQLPNLPRD- : 116
Cgla   : EEEAG--LTKQIMEYYI-------------------------EISD----ESEDKIEQLSLPQI- : 114
Ecy    : ISKED-DTVRKPIEFYL-------------------------ELDN------LVNEID------- : 107
Ego    : VPSEEDNTVRKPIEFYL-------------------------ELSD--------SATGFELIE-- : 111
Lth    : QDSEE--SIRAPVELYI-------------------------EQGE------PEGINLPDIE--- : 109
Tph    : EDEND--MVRKSMELYI------------------------DVDKD------STATNLIELPKID : 114
Vpo    : EGDEDSNQFRKSLELYI-------------------------AVVS------DKSDDYIKLPSIE : 115
Nca    : EGNEETGMVRKPLELYI-------------------------NVTD------DNSIKLTLPPID- : 111
Nda    : EENDA-GMCRKPLELYL-------------------------ELRN---EISGQNIPKIILPELD : 114
Sce    : EGDTM---IRKAIELFL-------------------------DVDD------ELPCNTCSLPDVE : 112
Tde    : EGVDG-GSYRKAIELYI-------------------------ETVK------GPLENEINLPDVV : 114
Zro    : EGDES-EMYRKPLEIYL-------------------------DVVE-----ESAINSEIELPALE : 115
```

Supplementary Figure S3. **Protein sequence alignment of the Sae2 UFD domain from metazoa and Saccharomycetales.** Human Sae2 homolog sequences were retrieved from EggNOG database. After removal of incomplete sequences or sequences exceeding in length, 86 sequences were retained. Selected sequences were aligned using MUSCLE at EMBL-EBI. The sequence alignment fragment corresponding to the LHEB2 domain is enclosed in a red rectangle. Residue shading correspond to 90% (white letter and dark background), 70% (white letter and gray background), and 50% (black letter and light gray background) of sequence identity.

Phylum	Symbol	Sequence code	Species
Arthropoda	Aae	>7159.AAEL010641-PA	Aedes aegypti (yellow fever mosquito), species, mosquitos
Arthropoda	Ace	>12957.ACEP_00011683-PA	Atta cephalotes (Leafcutter ant)
Arthropoda	Aga	>7165.AGAP008637-PA	Anopheles gambiae (African malaria mosquito), species, mosquitos
Arthropoda	Ame	>7460.GB12173-PA	Apis mellifera (honey bee), species, bees
Arthropoda	Api	>7029.ACYPI006138-PA	Acyrthosiphon pisum (pea aphid), species, aphids
Arthropoda	Cqu	>7176.CPIJ007700-PA	Culex quinquefasciatus (southern house mosquito), species, mosquitos
Arthropoda	Dgr	>7222.FBpp0149193	Drosophila grimshawi (species, flies)
Arthropoda	Dme_a	>7227.FBpp0076457	Drosophila melanogaster (fruit fly), species, flies
Arthropoda	Dpl	>13037.EHJ64540	Danaus plexippus (Monarch butterfly)
Arthropoda	Dps	>7237.FBpp0276753	Drosophila pseudoobscura (species, flies)
Arthropoda	Dpu	>6669.DappuP300104	Daphnia pulex (common water flea), species, crustaceans
Arthropoda	Dvi	>7244.FBpp0226639	Drosophila virilis (species, flies)
Arthropoda	Dwi	>7260.FBpp0249132	Drosophila willistoni (species, flies)
Arthropoda	Dya_a	>7245.FBpp0265382	Drosophila yakuba (species, flies)
Arthropoda	Dan	>7217.FBpp0113673	Drosophila ananassae (species, flies)
Arthropoda	Tca	>7070.TC002294-PA	Tribolium castaneum (red flour beetle), species, beetles
Arthropoda	Bmo	>7091.BGIBMGA012116-TA	Bombyx mori (domestic silkworm), species, moths
Arthropoda	Hme	>34740.HMEL017289-PA	Heliconius melpomene
Arthropoda	Nvi	>7425.NV10104-PA	Nasonia vitripennis (jewel wasp), species, wasps &c
Arthropoda	Phu	>121225.PHUM280660-PA	Pediculus humanus
Chordata	Aca	>28377.ENSACAP00000001485	Anolis carolinensis (Green anole) (American chameleon)
Chordata	Amel	>9646.ENSAMEP00000017826	Ailuropoda melanoleuca (giant panda), species, carnivores
Chordata	Bta	>9913.ENSBTAP00000003333	Bos taurus (cattle), species, even-toed ungulates
Chordata	Cjac	>9483.ENSCJAP00000020075	Callithrix jacchus (white-tufted-ear marmoset), species, primates
Chordata	Clu	>9615.ENSCAFP00000010792	Canis lupus familiaris (dog), subspecies, carnivores
Chordata	Cpo	>10141.ENSCPOP00000009465	Cavia porcellus (Guinea pig)
Chordata	Dre	>7955.ENSDARP00000102182	Danio rerio (zebrafish), species, bony fishes
Chordata	Eca	>9796.ENSECAP00000000109	Equus caballus (horse), species, odd-toed ungulates
Chordata	Fca	>9685.ENSFCAP00000020786	Felis catus (domestic cat), species, carnivores
Chordata	Gac	>69293.ENSGACP00000004237	Gasterosteus aculeatus (three-spined stickleback), species, bony fishes
Chordata	Gga	>9031.ENSGALP00000038502	Gallus gallus (chicken), species, birds
Chordata	Gmo	>8049.ENSGMOP00000004426	Gadus morhua (Atlantic cod), species, bony fishes
Chordata	Hsa	>9606.ENSP00000246548	Homo sapiens
Chordata	Itr	>43179.ENSSTOP00000002332	Ictidomys tridecemlineatus (thirteen-lined ground squirrel), species, rode
Chordata	Laf_a	>9785.ENSLAFP00000011542	Loxodonta africana (African savanna elephant), species, placentals
Chordata	Mga	>9103.ENSMGAP00000006132	Meleagris gallopavo (turkey), species, birds
Chordata	Mlu_a	>59463.ENSMLUP00000002773	Myotis lucifugus (little brown bat), species, bats
Chordata	Mlu_b	>59463.ENSMLUP00000018571	Myotis lucifugus (little brown bat), species, bats
Chordata	Mmul	>9544.ENSMMUP00000007742	Macaca mulatta (Rhesus monkey), species, primates
Chordata	Mmus	>10090.ENSMUSP00000099807	Mus musculus
Chordata	Mpu	>9669.ENSMPUP00000007408	Mustela putorius furo (domestic ferret), subspecies, carnivores
Chordata	Nle	>61853.ENSNLEP00000013124	Nomascus leucogenys ((northern white-cheeked gibbon), species, primates)
Chordata	Ocu	>9986.ENSOCUP00000001260	Oryctolagus cuniculus (rabbit), species, rabbits & hares
Chordata	Ola	>8090.ENSORLP00000015752	Oryzias latipes (Japanese medaka), species, bony fishes
Chordata	Oni	>8128.ENSONIP00000013939	Oreochromis niloticus (Nile tilapia), species, bony fishes
Chordata	Pca	>9813.ENSPCAP00000009534	Procavia capensis (Cape rock hyrax), species, placentals
Chordata	Ptr	>9598.ENSPTRP00000018512	Pan troglodytes (chimpanzee), species, primates
Chordata	Rno	>10116.ENSRNOP00000028672	Rattus norvegicus
Chordata	Sha	>9305.ENSSHAP00000002779	Sarcophilus harrisii (Tasmanian devil), species, marsupials
Chordata	Tgu_a	>59729.ENSTGUP00000009909	Taeniopygia guttata (zebra finch), species, birds
Chordata	Tni	>99883.ENSTNIP00000005165	Tetraodon nigroviridis (spotted green pufferfish), species, bony fishes
Chordata	Xma	>8083.ENSXMAP00000016139	Xiphophorus maculatus (southern platyfish), species, bony fishes
Chordata	Cin	>7719.ENSCINP00000030774	Ciona intestinalis ((vase tunicate), species, tunicates)
Chordata	Lcha	>7897.ENSLACP00000007856	Latimeria chalumnae (coelacanth), species, coelacanths
Chordata	Mdo	>13616.ENSMODP00000014955	Monodelphis domesticus
Chordata	Oan	>9258.ENSOANP00000015209	Ornithorhynchus anatinus (platypus), species, monotremes
Chordata	Oga	>30611.ENSOGAP00000007933	Otolemur garnettii (Small-eared galago) (Garnett's greater bushbaby)
Chordata	Psi	>13735.ENSPSIP00000010107	Pelodiscus sinensis
Chordata	Tru	>31033.ENSTRUP00000016721	Takifugu rubripes (Japanese pufferfish) (Fugu rubripes)
cnidaria	Nve	>45351.NEMVEDRAFT_v1g161397-PA	Nematostella vectensis ((starlet sea anemone), species, sea anemones)
cnidaria	Hma	>6085.XP_002165708	Hydra magnipapillata
Nematoda	Cbr	>135651.CBN10217	Caenorhabditis brenneri (Nematode worm)
Nematoda	Cbri	>6238.CBG13604	Caenorhabditis briggsae (nematodes)
Nematoda	Cel	>6239.W02A11.4	Caenorhabditis elegans (roundworm), species, nematodes
Nematoda	Cjap	>281687.CJA09929	Caenorhabditis japonica
Nematoda	Cre	>31234.CRE28085	Caenorhabditis remanei (Caenorhabditis vulgaris)
Platyhelminthes	Sma	>6183.Smp_166220__mRNA	Schistosoma mansoni (flatworns)
Porifera	Aqu	>400682.PAC_15728046	Amphimedon queenslandica (species, sponges)

Supplementary Table S1. *Phylum, symbol, sequence code and species used in the alignment of the Sae2 UFD domain from metazoa*.
